# Selective sweep and GWAS provide insights into adaptive variation of *Populus cathayana* leaves

**DOI:** 10.48130/forres-0024-0009

**Published:** 2024-04-09

**Authors:** Xinglu Zhou, Xiaodong Xiang, Demei Cao, Lei Zhang, Jianjun Hu

**Affiliations:** 1 State Key Laboratory of Tree Genetics and Breeding, Key Laboratory of Tree Breeding and Cultivation of National Forestry and Grassland Administration, Research Institute of Forestry, Chinese Academy of Forestry, Beijing 100091, China; 2 Co-Innovation Center for Sustainable Forestry in Southern China, Nanjing Forestry University, Nanjing 210037, Jiangsu, China

**Keywords:** *Populus cathayana*, Leaf traits, Selective sweep, GWAS

## Abstract

Leaf morphology plays a crucial role in predicting the productivity and environmental adaptability of forest trees, making it essential to understand the genetic mechanisms behind leaf variation. In natural populations of *Populus cathayana*, leaf morphology exhibits rich intraspecific variation due to long-term selection. However, there have been no studies that systematically reveal the genetic mechanisms of leaf variation in *P. cathayana*. To fill this gap and enhance our understanding of leaf variation in *P. cathayana*, we collected nine leaf traits from the *P. cathayana* natural population, consisting of 416 accessions, and conducted the preliminary classification of leaf types with four categories. Subsequently, we conducted an analysis of selective sweep and genome-wide association studies (GWAS) to uncover the genetic basis of leaf traits variation. Most of the leaf traits displayed significant correlations, with broad-sense trait heritability ranging from 0.38 to 0.74. In total, three selective sweep methods ultimately identified 278 positively selected candidate regions and 493 genes associated with leaf size. Single-trait and multi-trait GWAS methods detected 13 and 59 genes, respectively. By integrating the results of selective sweep and GWAS, we further identified a total of nine overlapping genes. These genes may play a role in the leaf development process and are closely associated with leaf size. In particular, the gene *CBSCBSPB3* (Pca07G009100) located on chromosome 7, was associated with the response to light stimulation. This study will deepen our understanding of the genetic mechanism of leaf adaptive variation in *P. cathayana* and provide valuable gene resources.

## Introduction

Leaf morphology plays a pivotal role throughout various stages of plant growth and development, influencing crucial processes such as gas exchange and transpiration^[1]^. Moreover, it serves as a significant indicator for plant identification and a predictive marker for productivity, being directly and quantifiably influenced by environmental changes^[2]^, and it also displays a wealth of variations. These variations are closely related to genetic variation and environmental factors. Understanding the genetic architecture and adaptive mechanisms of leaf variation has important ecological and evolutionary implications^[3]^. Currently, our understanding of the leaf trait variation in forest trees still requires further research.

Common garden experiments are the most common approach used to study the genetic variation of traits in forest tree populations. Typically, these studies combine phenotypes and genomic variation to dissect genetic diversity^[3−6]^. Compared to other tree species, poplars exhibit rapid growth, are easy to propagate asexually, and have a relatively mature genetic transformation system^[7]^. Therefore, poplars are generally considered as model species for forest tree research^[8]^. Previous studies have identified several quantitative trait loci (QTLs) and single nucleotide polymorphisms (SNPs) associated with morphological traits of poplar leaves^[4,8,9]^. On this basis, a series of studies have been conducted on leaf variation in tree species such as *Coffea mauritiana*^[10]^, *Ginkgo biloba*^[11]^, and *Camellia sinensis*^[12]^. These studies have provided preliminary explorations into the genetic basis of leaf trait variation and identified candidate genes associated with leaf color, morphology, and thickness. They provide initial insights into the variation of leaf traits in forest trees controlled by multiple genes, but further exploration is still required.

With advances in molecular biology, association genetics has emerged as a powerful tool for identifying the loci or genes underlying traits of interest. Among them, genome-wide association studies (GWAS) have been particularly prominent, playing an important role in understanding the genetic basis of plant traits^[13]^. Despite the high heritability of many morphological traits in forest trees, the significant SNPs associated with them only explain a small proportion of the heritability^[14,15]^. Complex traits often exhibit polygenicity, and traditional GWAS often struggle to detect the effects of alleles with small effect and low frequency^[16,17]^. Multi-trait GWAS overcomes this problem to some extent, using the correlations among traits and the combined weak genetic effects across traits to enhances GWAS statistical power and the ability to detect novel SNP loci^[15,18]^. Currently, the commonly used models for multi-trait GWAS are mainly the multivariate linear mixed model (mvLMM) and multi-trait mixed model (MTMM)^[19−21]^. Among these, the mvLMM model runs faster than the MTMM and has higher accuracy for groups with smaller sample sizes^[19]^, making it more suitable for forest tree studies. So far, it has been widely used in several tree species such as *Populus trichocarpa*^[4,8]^, *Populus deltoide*s^[22]^, and *Sequoiadendron giganteum*^[23]^. Among them, Chhetri et al.^[4]^ identified four and 20 candidate genes using single-trait and multi-trait GWAS, respectively, in their study of leaf traits in *P. trichocarpa*; Xia et al.^[24]^ detected five and 114 significant associations using single-trait and multi-trait GWAS, respectively, in their research on growth traits of perennial hybrid *Liriodendron*. Indeed, multi-trait analyses can increase power not only to detect pleiotropic genetic variants, but also genetic variants that affect only one of multiple correlated phenotypes^[25]^. Multi-trait and single-trait GWAS should be viewed as complementary rather than competitive^[19]^, and their integration will be beneficial for GWAS mining.

*Populus cathayana* is a native species in China with rapid growth and ability to adapt to various complex environments^[26,27]^. The leaf morphology of *P. cathayana* population has shown a rich genetic variation. Here, we first conducted a study on the whole-genome variation of *P. cathayana* leaves traits. Based on the phenotypic data from a common garden experiment, we performed selective sweep and GWAS analysis to detect the genetic basis of leaf variation in *P. cathayana*. The aim is to deepen our understanding of leaf adaptive evolution and identify potential candidate genes. These findings will provide preliminary insights for the adaptive variation of forest leaves, and valuable genetic resources for poplar improvement.

## Materials and methods

### Plant materials and phenotype collection

The *Populus cathayana* Rehder (Salicaceae, Tacamahaca) association population consists of 416 genotypes, collected from 34 natural distribution areas in China^[28]^, spaced at least 100 m from each other. All genotypes were propagated through vegetative reproduction by cuttings in the greenhouse of the Chinese Academy of Forestry. After two months of growth, they were transplanted to a common garden in Beijing, with 10−20 seedlings per genotype. The seedlings were spaced at 30 cm × 50 cm, and uniform water and fertilizer management was implemented.

### Phenotype data collection

In the spring of 2018, all *P. cathayana* genotypes were pruned at a height of 3 cm above the ground. In mid-July of the same year, three healthy and insect-free saplings were selected from each genotype, and fully expanded leaves from the 7^th^, 8^th^, and 9^th^ positions counting from the top were collected to measure leaf chlorophyll content and morphological indices. The relative chlorophyll content (SPAD) of the leaves was determined using a hand-held Soil and Plant Analyzer Development chlorophyll meter, and the leaves were scanned and imaged using a scanner. After that, the leaf area (LA), leaf perimeter (LP), leaf length (LL), leaf width (LW) and petiole length (PL) were measured using Digimizer image processing software. Three compound leaf morphology traits were calculated according to the method described by Cheng et al.^[29]^.

$ Leaf\;index\left(LI\right) =Leaf\;length/Leaf\;width $

$ Relative\;petiole\;length\left(RPL\right) =Petiole\;length/Leaf\;length $

$ Leaf\;margin\;factor\left(LMF\right) = 4*Leaf\;area/{Leaf\;perimeter}^{2} $

### Statistical analyses

All measurements were checked for recording errors, and outliers were removed. Phenotypic descriptive statistics (Supplemental Table S1) were calculated using dplyr v.1.1.2^[30]^. The variance components were estimated by applying a mixed linear model using lme4 v.1.1.34^[31]^, with genotype as a random factor and replication as a fixed factor. The generalized heritability (H^2^) was calculated using the formula H^2^ = Vg/(Vg + Ve), where Vg and Ve represent the genetic and residual variance components, respectively. Additionally, based on the statistical results of leaf traits, we performed PCA analysis using FactoMineR v1.34^[32]^, and conducted hierarchical clustering analysis using ggtree v.3.4.4^[33]^.

### Genotype data information

Preparation of the genotypic data was as described in Xiang et al.^[34]^. Briefly, paired-end libraries were prepared according to Illumina's standard, and the *P. cathayana* association population was subjected to whole-genome resequencing using the Illumina HiSeq 2500 platform. Each sample was sequenced to a depth of approximately 30 X. After filtering the raw reads, the high-quality paired-end sequencing reads were aligned to the reference genome of *P. cathayana* (https://ngdc.cncb.ac.cn/?lang=en, PRJCA014016) using the BWA-MEM algorithm from the BWA v.0.7.8^[35]^. Subsequently, PCR amplified repeats were removed using SAMtools v.0.1.19^[36]^, and SNP detection was performed using Genome Analysis Toolkit (GATK) v.4.0.4.0^[37]^. Finally, SNPs with an average sequencing depth < 20, quality < 30, minor allele frequency < 0.05, and missing genotype > 0.05 were filtered out using VCFtools v.0.1.16^[38]^.

### Selective sweep analysis

Selective sweep helps to identify genomic information associated with adaptive variations. Here, we used three methods for selective sweep analysis. Genetic differentiation coefficient (*Fst*) and nucleotide diversity (*π*) were calculated using the sliding window method with VCFtools v.0.1.16. XP-CLR was performed using the python version of XP-CLR v.1.1.2^[39]^ with the parameters: --ld 0.95 -maxsnps 600 --size 10 kb --step 1 kb. Referring to the studies by Li et al.^[3]^ & San et al.^[40]^, we similarly used the top 5% window as the candidate region for selective scanning analysis. The overlapping regions among the three methods were defined as the final candidate regions for selective sweep. Then, the genes within final candidate regions were defined as selective sweep candidate genes, and enrichment analysis was performed on these selective sweep candidate genes using the GO (Gene Ontology) and KEGG (Kyoto Encyclopedia of Genes and Genomes) databases.

### Genome-wide association studies (GWAS)

To further identify genes that play a key role in the variation of *P. cathayana* leaf traits, we conducted GWAS analysis. We first filtered out SNPs with a minor allele frequency > 0.05 and a missing genotype rate < 0.05 using VCFtools v.0.1.16^[38]^. Subsequently, we used Plink v1.90^[41]^ (window size 50, step size 50, r^2^ ≥ 0.20) to filter out SNPs with a high degree of linkage disequilibrium to ensure the independence of the SNPs. Ultimately, 587,765 SNPs were retained for association analysis. We first conducted single-trait GWAS (Supplemental Table S2) using univariate linear mixed model (LMM) in GEMMA v.0.98.3^[42]^. In order to eliminate false positives in association analysis, the first three principal components (Supplemental Table S3) were used as a fixed effect, while kinship matrix (Supplemental Table S4) was used as a random effect. Principal component analysis (PCA) was performed using GCTA v1.93.2. Kinship matrix was also constructed using GEMMA v.0.98.3. The GWAS model was:

$ \mathrm{y}=\mathrm{W}\mathrm{\alpha }+\mathrm{x}\beta +\mathrm{u}+\mathrm{e}, $

where y is a vector of quantitative traits for *n* individuals, W is an *n × c* matrix of covariates (fixed effects), α is a vector of corresponding coefficients including the intercept, x is an *n*-vector of marker genotypes, β is the effect size of the marker, u is an *n*-vector of random effects, and e is an *n*-vector of errors^[42]^.

### Multi-trait GWAS

Multi-trait GWAS enhances the signal strength of SNPs to some extent, and the degree of enhancement is closely associated with the correlation and functional relationships between traits. Here, we constructed five trait combinations (Supplemental Table S5) and employed the multivariate linear mixed model (mvLMM) in the GEMMA v0.98.3 to perform multi-trait GWAS. For example, based on inter-trait correlations, we combined four closely correlated leaf morphological traits (leaf area, leaf circumference, leaf length, and leaf width) as the main structural units of leaves. Considering the functional relationships among traits, we combined leaf area, SPAD and petiole length^[4]^ as they may have potential effects on leaf photosynthesis. Considering the impact of leaf index and leaf margin factor on understanding leaf morphological traits and their implications for plant adaptability^[43]^, we constructed two trait combinations based on the sub-traits of these two composite traits. In addition, we combined the three composite traits (relative petiole length, leaf index and leaf margin factor) as the overall evaluation of leaf traits. The multi-trait GWAS model was:

$ \mathrm{Y}=\mathrm{W}\mathrm{A}+\mathrm{x}{\beta }^{d}+\mathrm{U}+\mathrm{E}, $

where Y is an *n* by *d* matrix of quantitative traits for *n* individuals, W is an *n × c* matrix of covariates (fixed effects), A is *c* by *d* matrix of corresponding coefficients including the intercept, x is an *n*-vector of marker genotypes, *β*^*d*^ is a *d* vector of marker effect sizes for the *d* phenotypes, U is an *n* by *d* matrix of random effects, and E an *n* by *d* matrix of errors^[42]^.

### Analyses of GWAS results

CMplot v4.3.1 was used to generate Q-Q plot to assess the accuracy of the model and create Manhattan plots to visualize the GWAS results^[44]^. We used a *p*_value cutoff based on the Bonferroni correction criterion of 8.51 × 10^−8^ (0.05/*n*, where *n* is the effective number of SNPs) to identify significant associations for single-trait and multi-trait GWAS. Additionally, for single-trait GWAS, we also used a more liberal *p*_value cutoff of 1.70 × 10^−6^ (1/*n*, where *n* is the effective number of SNPs) to identify suggestive associations. For significant associations, we used the reference genome of *P. cathayana* as a reference and considered their upstream and downstream regions of 20 kb as candidate intervals to obtain GWAS candidate genes. Percentage of variance explained (PVE) by SNPs was estimated using the formula described by Shim et al.^[45]^. Since multi-trait output does not provide SE for effect size, the estimation of PVE for SNPs was not conducted. The PVE calculation formula was:

$ PVE =\dfrac{{2\beta }^{2}{MAF(1-MAF)}}{{2\beta }^{2}{MAF(1-MAF)+}{\left(se\right(\beta \left)\right)}^{2}{2}{NMAF(1-MAF)}} $

where, β = effect size, MAF = minor allele frequency, N = sample size, se(β) = SE of β.

### Candidate gene functional enrichment and network analysis

Based on the gene annotation information of the *P. cathayana* genome, we used the GO and KEGG databases to predict the potential biological functions of candidate genes. To further screen candidate genes, we constructed a candidate gene network using Cytoscape v3.7.1^[46]^. Nodes denote traits and genes and node size denotes connectivity.

## Results

### Correlations and variation analysis of leaf traits

**Figure 1:**
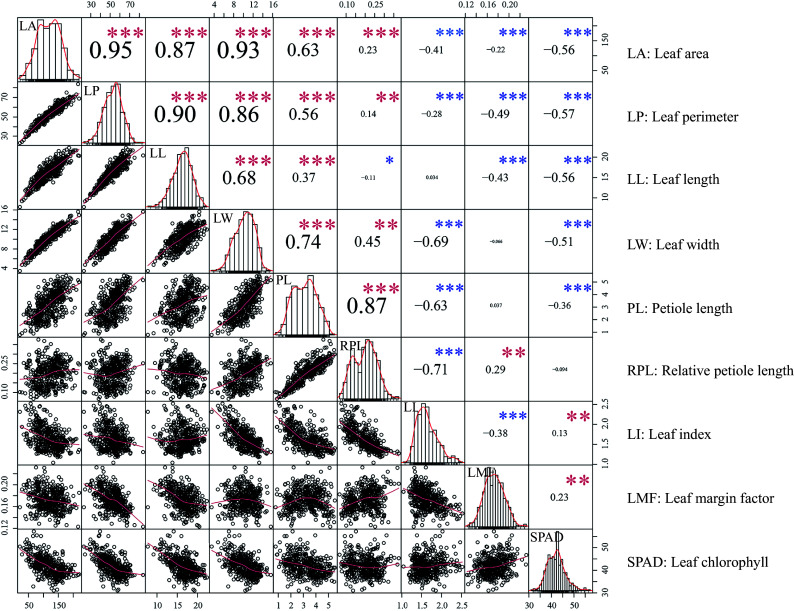
Relationships between leaf traits. Scatter plots and histograms display the distribution of each trait. The red line overlaid on each histogram represents the density distribution. '*', '**' and '***' denote significant correlations at the 0.05, 0.01, and 0.001 levels, respectively. Red and blue asterisks represent positive and negative correlations, respectively.

The distribution of leaf traits and correlations analysis between traits are shown in Fig. 1. Most leaf traits were significantly different (*p* < 0.01) within the *P. cathayana* population. The same results were also found between different source regions^[28]^. The five leaf morphological traits were significantly positively correlated among each other, and significantly negatively correlated with two composite traits (LI and LMF) as well as SPAD (Fig. 1). Broad-sense heritabilities ranged from 0.3791 to 0.7375 for leaf traits, with the highest being LMF and the lowest being SPAD coefficient of variation (Table 1).

**Table 1:** Results of statistical analysis of leaf traits.

Trait	Abbreviation	Mean	SD	Min	Max	CV^a^	Vg^b^	Ve^c^	H^2d^
Leaf area	LA	118.272	37.235	21.034	219.587	0.31	1,143.7214	685.7488	0.6252
Leaf perimeter	LP	51.986	9.642	23.017	83.762	0.19	87.9111	66.3378	0.5699
Leaf length	LL	16.344	2.511	7.733	22.327	0.15	5.4303	2.7019	0.6678
Leaf width	LW	10.242	2.072	3.483	15.633	0.20	3.9290	2.9150	0.5741
Petiole length	PL	3.205	1.047	0.786	5.513	0.33	0.9876	0.3814	0.7214
Relative petiole length	RPL	0.197	0.061	0.066	0.376	0.31	0.0671	0.0256	0.7234
Leaf index	LI	1.645	0.272	1.042	2.520	0.17	0.0003	0.0005	0.3791
Leaf margin factor	LMF	0.173	0.019	0.123	0.232	0.11	0.0033	0.0012	0.7375
Leaf chlorophyll	SPAD	41.989	4.587	30.993	57.233	0.11	7.0770	4.9680	0.5875
^a^ Coefficient of variation; ^b^ genetic variance; ^c^ residual variance; ^d^ heritability,									

### PCA and cluster analysis of leaf phenotypic data in *P. cathayana* population

**Figure 2:**
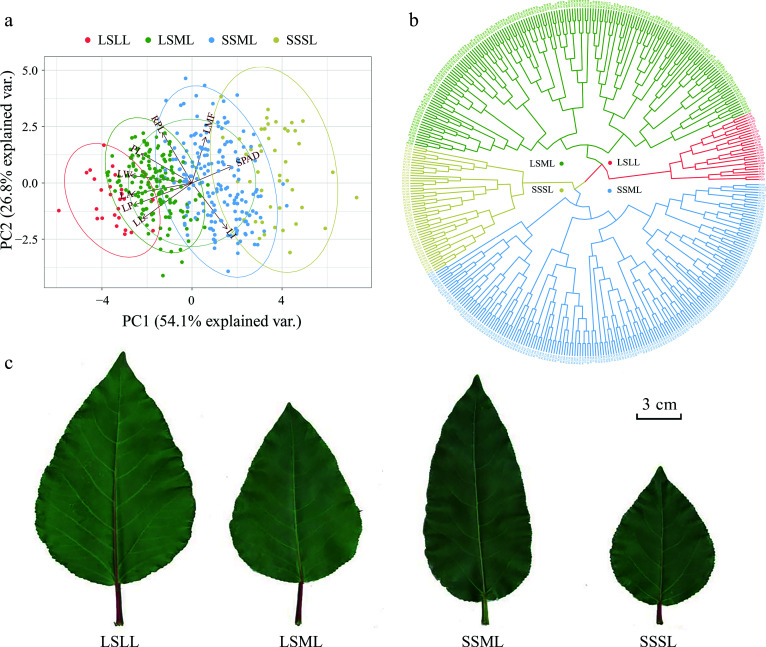
Principal component analysis (PCA) and hierarchical clustering analysis based on leaf phenotype data. (a) The PCA plot showing the first and second principal components of *P. cathayana* genotypes, represented by color-coded points, and the relative importance of explanatory variables is indicated by vectors. (b) Clustering of *P. cathayana* populations into four categories based on leaf traits. (c) Comparison of four leaf shape categories^[28]^.

We performed principal components analysis (PCA) to further explore the relationships among traits within the *P. cathayana* population. PC1 explained over 54% of the total variation, with PC1 and PC2 together explaining 80% of the total variation. Five morphological traits were negatively weighted towards the PC1 axis, while SPAD was positively weighted towards that axis. RPL and LMF were positively weighted towards the PC2 axis, while LI was negatively weighted towards that axis (Fig. 2a). Furthermore, through the clustering analysis, we have classified the *P. cathayana* leaves into four categories: long-stalked large leaf (LSLL), long-stalked medium leaf (LSML), short-stalked medium leaf (SSML), and short-stalked small leaf (SSSL) (Fig. 2b, c, Supplemental Table S6).

### Selective sweep identifies candidate genes regulating leaf morphology

**Figure 3:**
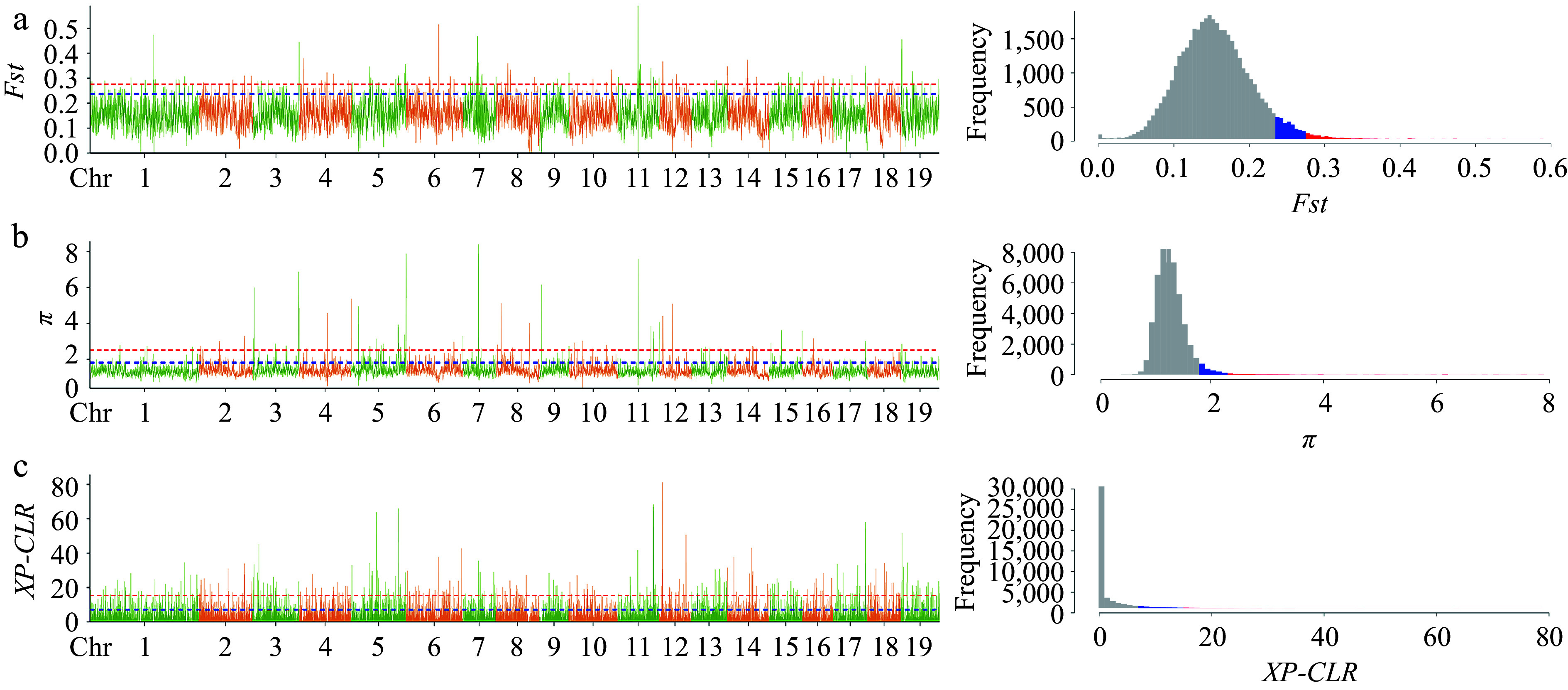
Selective sweep analysis visual line chart and frequency histogram. Genetic differentiation coefficient (*Fst*) and nucleotide diversity (*π*) were calculated by the sliding window method with a window size of 10 kb and a step size of 1 kb; *XP-CLR* has the same window size as *Fst* and *π*. The plots (a), (b) and (c) respectively represent the line graph and frequency histogram of *Fst*, *π*, and *XP-CLR*. In the line plot, the blue line and red line represent the top 5% and top 1% thresholds, respectively, and correspond to the blue and red shaded areas in the frequency histogram. The interval that passes the top 5% threshold lines in three methods is considered as the final candidate region for selective sweep.

**Figure 4:**
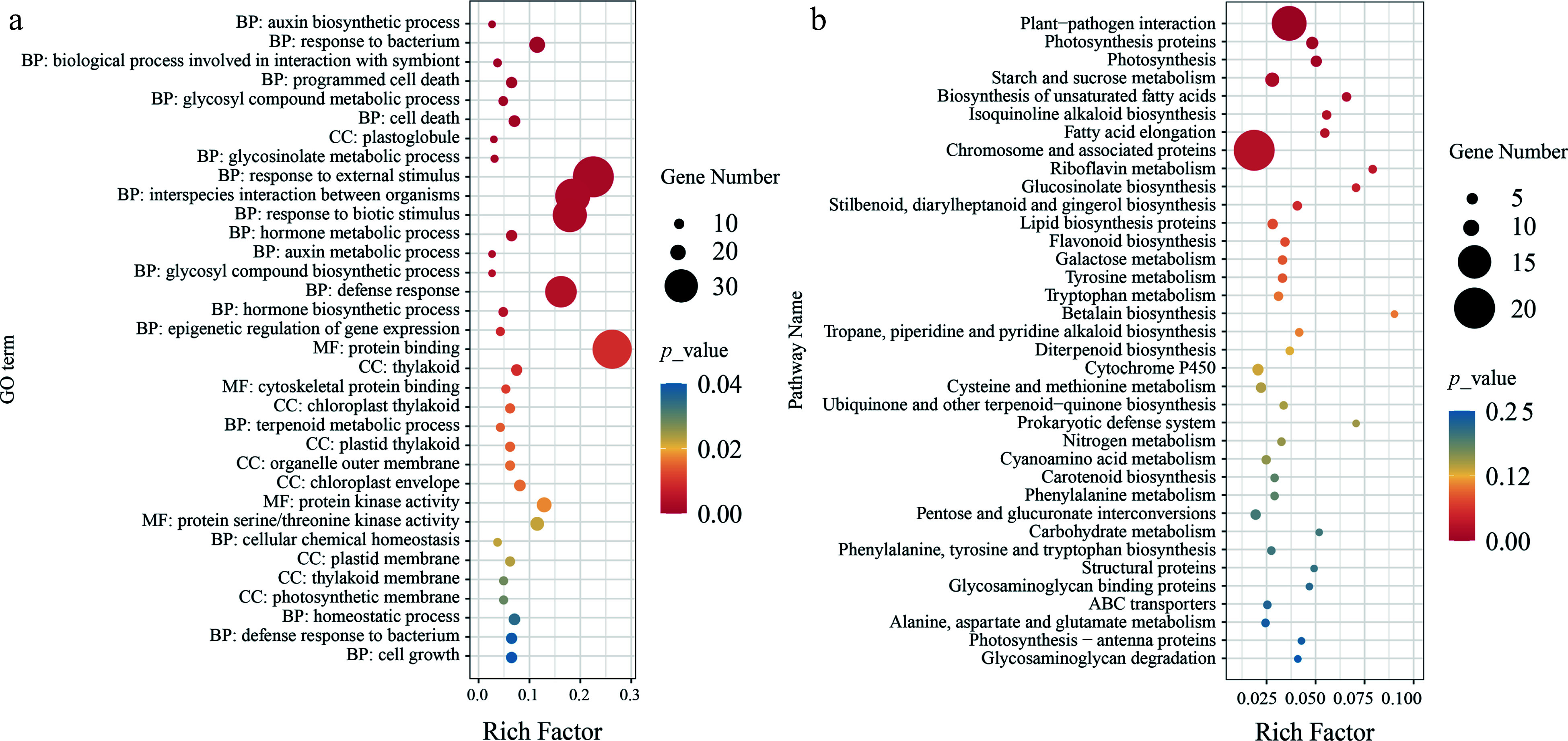
Functional enrichment analysis of selective sweep candidate genes. (a) GO enrichment analysis of candidate genes, where the size of the points represents the number of genes, and the color of the points indicates significance level. BP, CC, MF respectively represent biological process, cellular component, and molecular function. (b) KEGG enrichment analysis of candidate genes, where the size of the points represents the number of genes, and the color of the points indicates significance level.

In our previous study, we considered the *P. cathayana* population to be divided into four groups: NW (Northwest China), SW (Southwest China), TH (Tai-hang Mountains), and NC (North China) (Supplemental Fig. S1). We observed that LSLL genotypes were predominantly concentrated in the NW group, while SSSL genotypes were mainly found in the TH group. To identify selected genes associated with leaf size during leaf variation, we selected 10 LSLL genotypes from the TH group and 10 SSSL genotypes from the NW group for selective sweep analysis. *Fst*, *π*, and *XP-CLR* identified a total of 278 overlapping regions (Fig. 3). Further, 493 selective sweep candidate genes were identified (Supplemental Table S7). These candidate genes were significantly enriched in GO terms closely related to leaf development, including cell growth, cell death, auxin biosynthesis and metabolic processes, protein kinase activity, and aspects of the photosynthetic membrane such as chloroplast thylakoids (Fig. 4a, Supplemental Table S8). In addition, these genes were notably enriched in several metabolic pathways, including plant-pathogen interaction, photosynthesis, starch and sucrose metabolism, and biosynthesis of unsaturated fatty acids. Overall, enrichment analysis preliminarily suggests that the candidate genes play significant roles in leaf development (Fig. 4b, Supplemental Table S9).

### GWAS to identify candidate genes controlling leaf morphology

**Figure 5:**
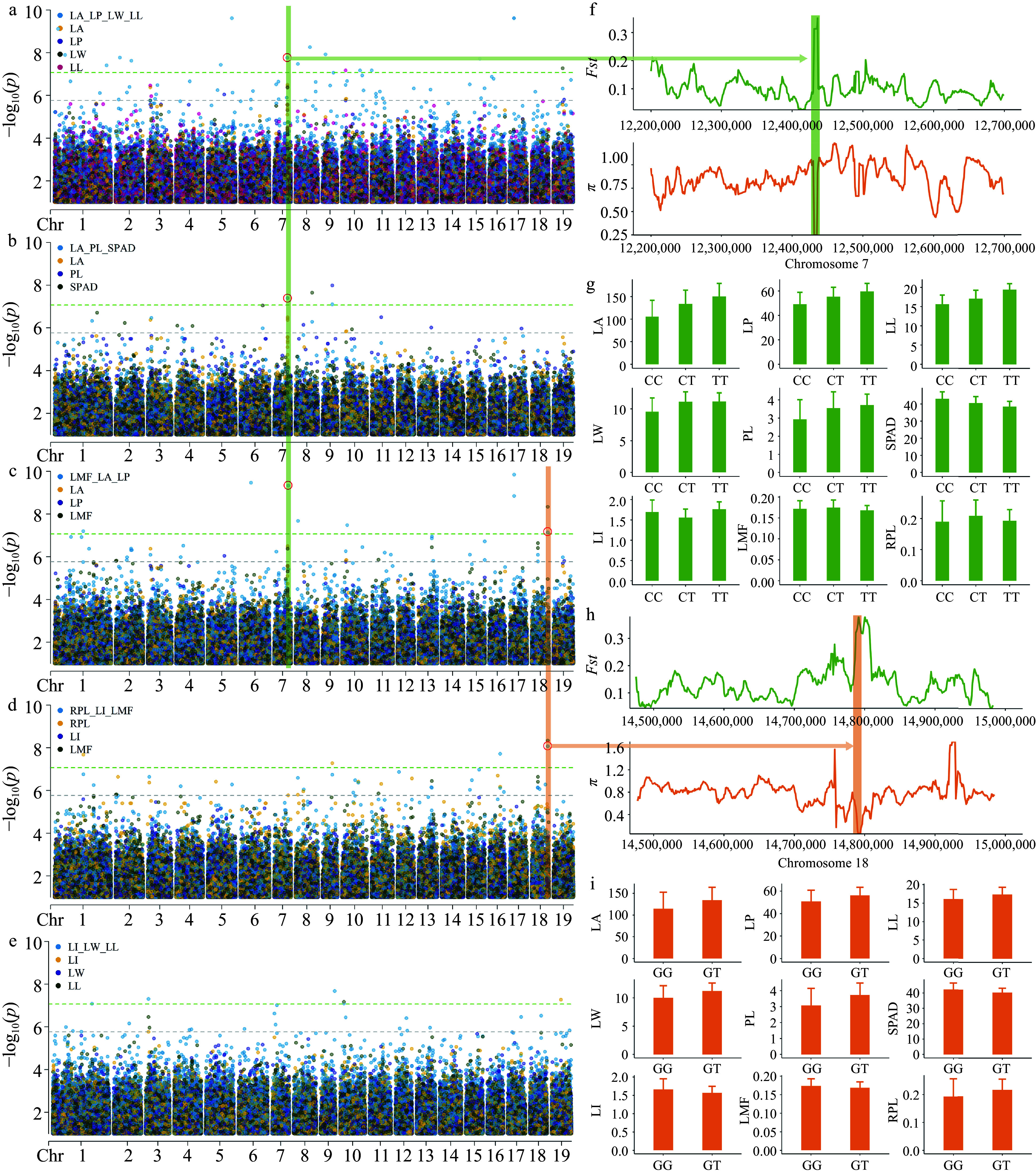
GEMMA multi-trait GWAS Manhattan plots and significant locus analysis. The plots (a), (b), (c), (d), and (e) correspond to the manhattan plots of LA_LP_LL_LW, LA_PL_SPAD, LI_LW_LL, LMF_LA_LP and RPL_LI_LMF, respectively. For ease of comparison, we have included the single-trait GWAS Manhattan plots of the traits in the figures. Each dot on the plot represents a SNP, with dot colors indicate single-trait or multi-trait associations. *p*_value were transformed into −log_10_(*p*_value). SNPs above green lines passed bonferroni correction test (*p* ≤ 8.51 × 10^−8^). The red circle represents the detected loci in multiple trait combinations. (f) Analysis of genetic differentiation (*Fst*) and nucleotide diversity (*π*) near SNP 7__12437614. (g) Comparison of leaf phenotypes corresponding to three haplotypes at SNP 7__12437614, represented by mean values in a bar graph with standard deviation as the error. (h) Analysis of genetic differentiation (*Fst*) and nucleotide diversity (*π*) near SNP 18__14798104. (i) Comparison of leaf phenotypes between two haplotypes at SNP 18__14798104, represented by mean values in a bar graph with standard deviation as the error.

GWAS was conducted to further identify candidate genes associated with leaf morphology. For single-trait GWAS, we identified a total of six significant SNPs across the nine leaf traits (Supplemental Figs S2 & 3). These SNPs were located within or near 13 gene, with explained PVE ranging from 1.08% to 3.34% (Table 2). For multi-traits GWAS, we identified a total of 33 significant SNPs associated with at least one of the five sets of traits (Fig. 5a−e, Supplemental Fig. S4). These SNPs were located within or close to 59 gene (Supplemental Table S10). Ultimately, the two GWAS methods identified a total of 67 candidate genes associated with leaf traits; Among them, five candidate genes overlapped in the two GWAS methods (Supplemental Table S11). In addition, using a more liberal *p*_value cutoff of 1.70 × 10^−6^, we associated a total of 41 suggestive association SNPs in nine single-trait GWAS (Supplemental Fig. S2). These SNPs explained PVE ranging from 0.01% to 3.18%, and identifying 52 suggestive candidate genes (Supplemental Table S12). Among these suggestive candidate genes, eight overlapped with the candidate genes from the multi-trait GWAS. These overlapping genes are initially deemed key candidate genes likely to influence the leaf morphology of *P. cathayana* (Supplemental Table S13).

**Table 2:** Statistical information of significant SNPs in single-trait GWAS.

rs^a^	Trait	*p*_value	PVE^b^	Gene	Functional annotation
19__8697977	LW	5.36E-08	3.15	Pca19G006210	Reverse transcriptase
				Pca19G006220	RNase H-like domain found in reverse transcriptase
10__3487289	LL	6.77E-08	2.97	Pca10G001830	Protein tyrosine and serine/threonine kinase
				Pca10G001840	D-mannose binding lectin
9__9068032	PL	1.04E-08	3.34	Pca09G008070	WD domain, G-beta repeat
				Pca09G008080	Clp protease
				Pca09G008090	C2H2-type zinc finger
8__15332723	SPAD	2.24E-08	3.24	Pca08G018800	5' nucleotidase family
1__26393650	RPL	2.10E-08	1.98	Pca01G022670	NA
				Pca01G022680	Protein of unknown function (DUF1117)
9__9068032	RPL	5.28E-08	2.20	Pca09G008070	WD domain, G-beta repeat
				Pca09G008080	Clp protease
				Pca09G008090	C2H2-type zinc finger
18__14798104	LMF	4.57E-09	1.08	Pca18G011690	Cytochrome P450
				Pca18G011700	Protein tyrosine and serine/threonine kinase
				Pca18G011710	Cytochrome P450
NA, not available. ^a^ The name of the SNP. ^b^ The proportion of phenotype variance explained by SNP.					

To further clarify the biological functions of the GWAS candidate genes, we conducted enrichment analysis on the GWAS candidate genes (Supplemental Fig. S5a, b). The candidate genes were involved in various GO terms, including saponin and glycoside biosynthetic and metabolic process, phytosteroid biosynthetic process, response to abscisic acid, response to stimulus, chloroplastic endopeptidase clp complex, response to hormone (Supplemental Table S14). Moreover, they are associated with KEGG metabolic pathways such as protein processing in endoplasmic reticulum, cyanoamino acid metabolism, phenylpropanoid biosynthesis, starch and sucrose metabolism, flavonoid biosynthesis, plant hormone signal transduction (Supplemental Table S15). The results of the enrichment analysis provide preliminary evidence that the GWAS candidate genes play a role in regulating leaf traits, either directly or indirectly.

### Identification of key loci regulating leaf variation

In multi-trait GWAS, there are two loci that show significant associations in different trait combinations, and they warrant further investigation. Among them, SNP 7__12437614 on chromosome 7 showed a close association with the LA trait (Fig. 5a−c), and was located within Pca07G009100 (*CBSCBSPB3*). This gene contains the CBS domain, and GO enrichment analysis indicates its involvement in the response to light stimulus. SNP 18__14798104 on chromosome 18 showed a close association with the LMF trait (Fig. 5c, d) and was located within the coding region of Pca18G011700 (*PIX13*). This gene encodes the tyrosine and serine/threonine kinase proteins, and GO enrichment analysis indicates its involvement in defense responses and responses to external stimuli, as well as other GO processes; The neighboring genes Pca18G011690 (*CYP716A17*) and Pca18G011710 both encode Cytochrome P450, which may be involved in the synthesis of chlorophyll and other photosynthetic pigments. *Fst* and *π* analysis results indicated significant differentiation between the SHSL and LHLL categories near these two SNPs (Fig. 5f, h). This further suggests that these loci may play a key role in promoting genetic differentiation and adaptive phenotypic variation. Furthermore, the observed phenotypic differences between different haplotypes provide support for the involvement of these SNPs in trait variation (Fig. 5g, i). These results further suggest that candidate genes may regulate leaf development by affecting leaf photosynthetic capacity.

### Candidate gene network analysis

**Figure 6:**
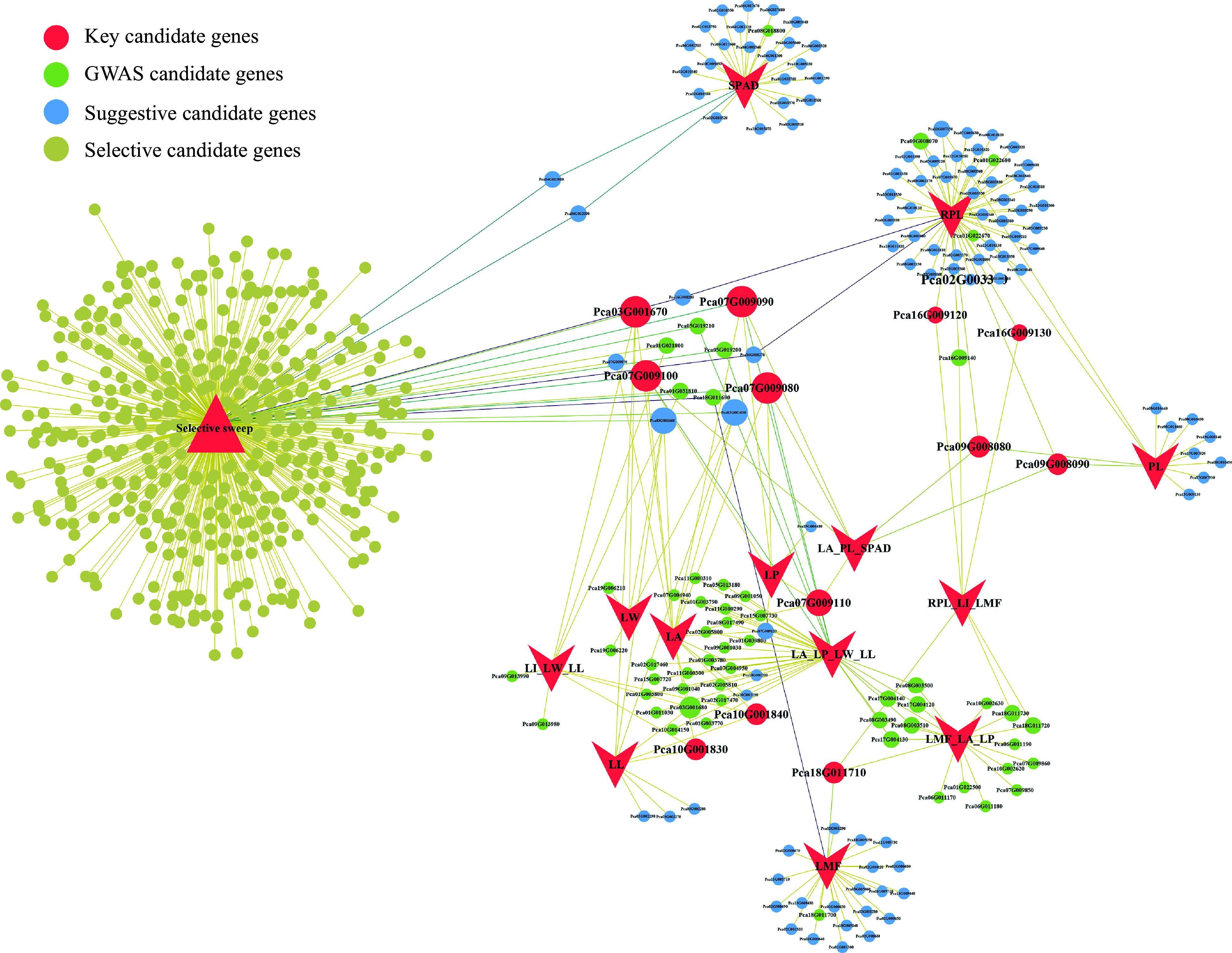
Candidate gene network visualization. Circles represent candidate genes, and the size of the circles indicates the number of associated traits. The color of the circles corresponds to different types of candidate genes. Lines represent the traits associated with genes and whether they are candidate genes for selective sweep.

To further identify important candidate genes, we constructed a candidate gene network using Cytoscape v3.7.1 (Fig. 6, Supplemental Table S16). The number of overlapping GWAS candidate genes among different leaf traits was closely related to the correlation among traits, with no overlapping genes found between SPAD and other traits. Within the entire network, 18 GWAS candidate genes were associated with at least two leaf trait variations. The gene Pca09G008080 (*CLPR1*), encoding an ATP-dependent Clp protease proteolytic subunit-related protein, and the gene Pca10G001830, encoding protein tyrosine and serine/threonine kinase, showed high connectivity in the network. Selective sweep and GWAS identified a total of nine overlapping genes (Supplemental Table S17), and these genes may play a role in the leaf development process and are closely associated with leaf size. Among them, the gene *CBSCBSPB3*, associated with the response to light stimulation, was identified as a key candidate gene in GWAS analysis of this study.

## Discussion

Leaf size and shape are crucial traits in the development and growth of poplar^[9]^. Identifying the genetic mechanisms underlying leaf trait variation holds profound implications for poplar breeding. For a long time, leaf trait research has been an important direction of forest tree research. However, our understanding of the gene regulatory networks and genetics underlying leaf development is still limited^[22,47]^. With advancements in biotechnology, the genomic era has provided possibilities for further studying the variations in leaf traits. *Populus*, as model forest tree species, has been subject to a series of GWAS studies on leaf traits, such as *P. trichocarpa*^[4]^ and *P. euphratica*^[48]^. These studies indicated that leaf traits have exhibited abundant genetic variation within poplar populations. Our study similarly confirms the presence of rich leaf variation in *P. cathayana*, enabling the preliminary classification of *P. cathayana* leaves into four categories (LSLL, LSML, SSML and SSSL).

Previous research has suggested that poplar leaf trait variations were primarily driven by geographical factors, with many leaf traits correlating with altitude and latitude^[8,49]^. *P. cathayana* leaf morphological traits exhibit high correlations among themselves and negative correlations with chlorophyll content (SPAD), which are consistent with previous studies. Furthermore, they also pointed out that the morphological characteristics of poplar leaves not only affect chlorophyll content but also influence leaf thickness and stomatal density, subsequently impacting poplar tree nitrogen content, stomatal conductance, and photosynthesis^[50]^, ultimately affecting tree growth. Significant variation in leaf traits within *P. cathayana* populations may be closely associated with environmental factors such as temperature, precipitation, and solar radiation lamps, in addition to genetic factors.

Previously, there have been no reports on whole-genome studies of leaf traits in *P. cathayana*. Here, we systematically carried out the first whole-genome study of *P. cathayana* leaves. We observed that the ability of GWAS to mine single traits was limited, and most traits were difficult to associate to specific genetic variant loci. This phenomenon is present not only for leaf traits but also for most traits of forest trees^[3,4,8,9,22,48,51]^. The reasons for this limitation have been discussed in previous studies^[4,14,52]^. One of the primary reasons is the relatively small population size used in forest tree association analyses, which makes it difficult to detect the effects of alleles with small effect sizes and low frequencies. Therefore, we considered applying multi-trait mixed linear model to improve the mining power in addition to the conventional GWAS analysis^[4,19]^. In our study, multi-trait GWAS identified more candidate genes than single-trait GWAS, and this increased power of multi-trait GWAS depends in part on the correlation among traits.

Selective sweep and GWAS provide possibility for studying adaptive variations and have been widely used^[53−57]^. Li et al.^[3]^ used GWAS, selective sweep and multi-omics data to identify 41 representative genes potentially involved in stomatal morphological variation and photosynthetic capacity in *P. tomentosa.* In this study, several genes (*CBSCBSPB3, CYP716A17, CLPR1*) were identified through selective sweep and GWAS, which may be related to leaf function in *Arabidopsis*^[58,59]^. Their functional annotations in the *P. cathayana* genome are associated with light stimulation, photosynthesis, chloroplast development, and stress response. Therefore, we preliminarily consider them to be key candidate genes for exploring leaf variation in *P. cathayana*. Furthermore, by selective sweep we also identified multiple genes associated with cell development, photosynthesis, and chloroplasts.

Compared to previously reported GWAS studies on poplar leaf traits^[3,4,9,22,48,51]^, we found few overlapping genes across different studies. By studying the functions of these candidate genes, we found that candidate genes are functionally enriched in cell development, carbohydrate metabolism, plant-pathogen interactions, and flavonoid biosynthesis. In our study, GWAS candidate genes also displayed similar functions. Notably, we found that unlike other leaf GWAS studies, *P. cathayana* leaf traits GWAS candidate genes were significantly enriched in GOs such as response to abscisic acid and mitotic cell cycle. We believe that our study provides valuable insights into a portion of the genetic variation behind the phenotypic traits of leaves.

## Conclusions

In summary, we have reported the first genome-wide study on leaf traits in *P. cathayana* and classified the leaf types of *P. cathayana* into four major categories. Integrating selective sweep and GWAS, we conducted a preliminary exploration of the genetic basis of leaf variation in *P. cathayana*. We detected candidate genes associated with photosynthesis and chloroplast development that may play a key role in leaf variation. Through single-trait and multi-trait GWAS analyses, we further identified candidate genes involved in regulating leaf morphology variation. Integrating selective sweep and GWAS result further identified the important role of *CBSCBSPB3* in leaf phenotype variations. Our findings advance the understanding of adaptive variation of leaf traits and provided valuable genetic resources for improving the adaptations of forest tree.

## SUPPLEMENTARY DATA

Supplementary data to this article can be found online.

## Data Availability

The *P. cathayana* genome sequencing data have been deposited in National Genomics Data Center (https://ngdc.cncb.ac.cn/?lang=en), under BioProject PRJCA014016, with accession numbers SAMC1029101-SAMC1029104. The whole-genome resequencing data have been deposited under National Genomics Data Center BioProject PRJCA014017 with accession numbers SAMC1029105-SAMC1029542. All other data related to this study can be located in the supplementary files of the article.
