# Supplementary material for: Selective sweep and GWAS provide insights into adaptive variation of Populus cathayana leaves
Source: For Res (Fayettev). 2024 Apr 9;4:e012. doi: 10.48130/forres-0024-0009 (PMC11524237; doi:10.48130/forres-0024-0009)
Supplement: Supplementary file 1 — Supplementary data to this article can be found online. [file forres-0024-0009-S1.zip › 10.48130_forres-0024-0009-Suppl-FigureS2.pdf]

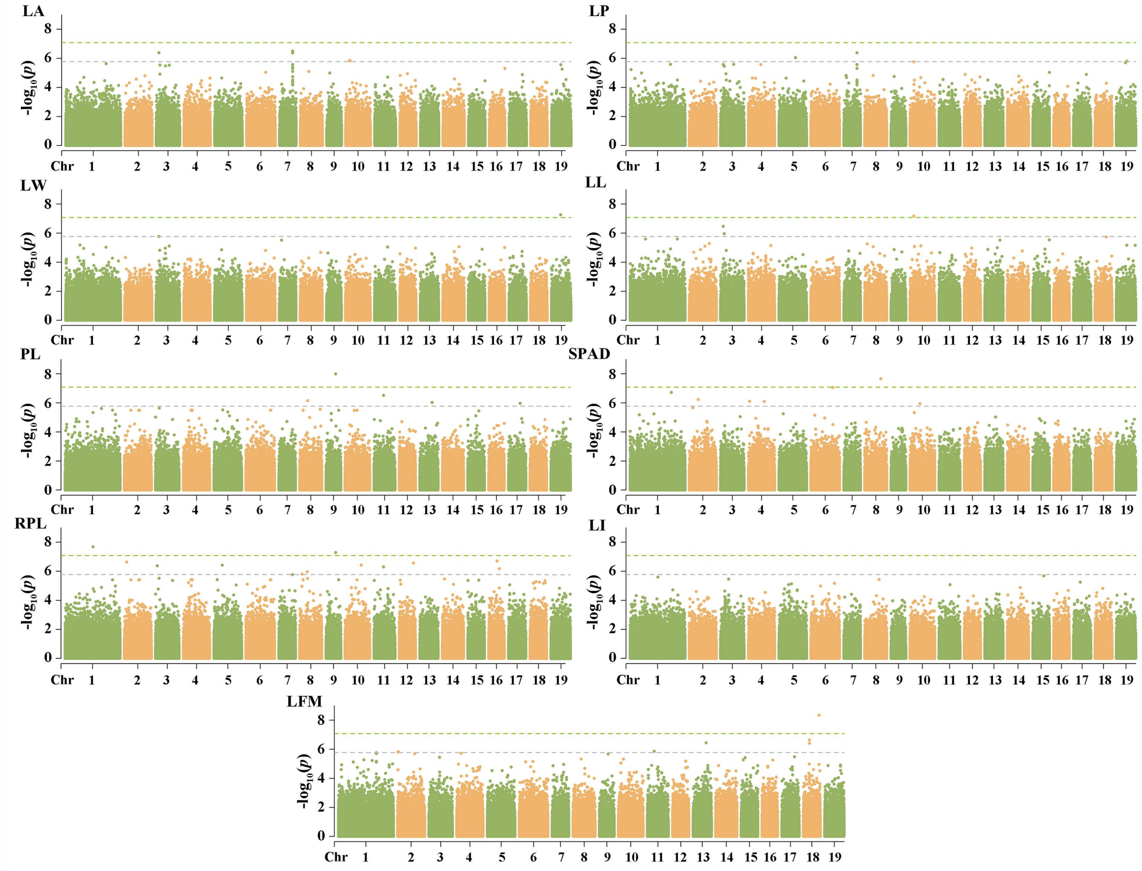

Supplementary Figure S2 Manhattan plots GEMMA single-trait GWAS in *P. cathayana*. *P-values* were transformed into $-\log_{10}(p)$. Each dot represents a single nucleotide polymorphism (SNP), and SNPs above green lines passed bonferroni correction test ($P \leq 8.51 \times 10^{-8}$), while SNPs above grey lines are considered suggestive associations ($P \leq 1.70 \times 10^{-6}$).
