# Supplementary material for: Selective sweep and GWAS provide insights into adaptive variation of Populus cathayana leaves
Source: For Res (Fayettev). 2024 Apr 9;4:e012. doi: 10.48130/forres-0024-0009 (PMC11524237; doi:10.48130/forres-0024-0009)
Supplement: Supplementary file 1 — Supplementary data to this article can be found online. [file forres-0024-0009-S1.zip › 10.48130_forres-0024-0009-Suppl-FigureS3.pdf]

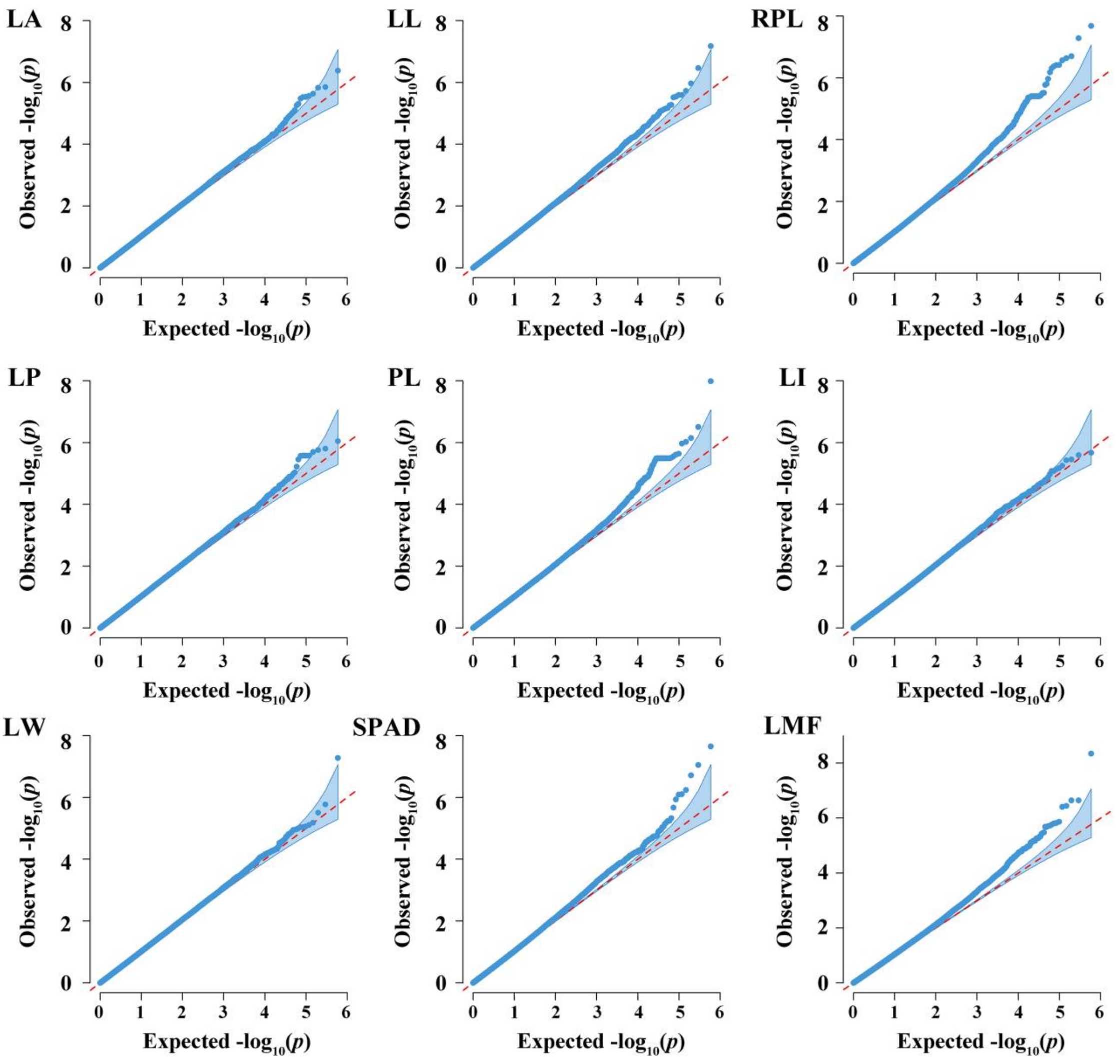

Supplementary Figure S3 QQ plot for single-trait GWAS analysis, corresponding to the manhattan plots for single-trait GWAS.
