# Supplementary material for: Selective sweep and GWAS provide insights into adaptive variation of Populus cathayana leaves
Source: For Res (Fayettev). 2024 Apr 9;4:e012. doi: 10.48130/forres-0024-0009 (PMC11524237; doi:10.48130/forres-0024-0009)
Supplement: Supplementary file 1 — Supplementary data to this article can be found online. [file forres-0024-0009-S1.zip › 10.48130_forres-0024-0009-Suppl-FigureS4.pdf]

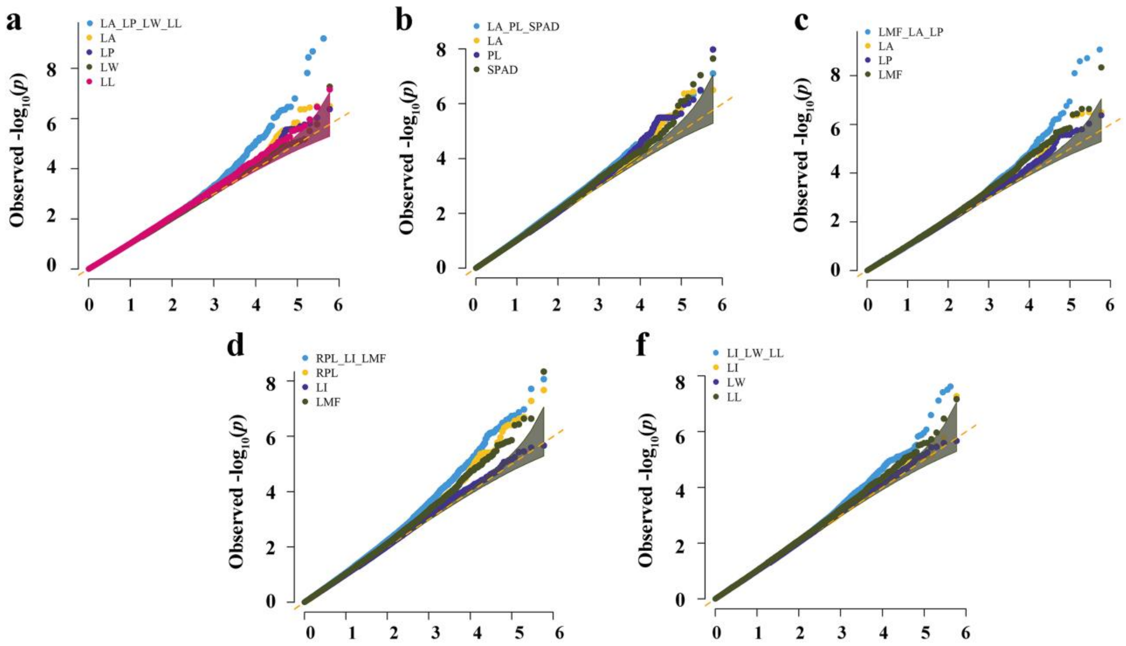

Supplementary Figure S4 QQ plot for multi-trait GWAS analysis. The plots (a, b, c, d, and e) correspond to the QQ plots of LA_LP_LL_LW, LA_PL_SPAD, LI_LW_LL, LMF_LA_LP and RPL_LI_LMF, respectively.
