# Supplementary material for: Selective sweep and GWAS provide insights into adaptive variation of Populus cathayana leaves
Source: For Res (Fayettev). 2024 Apr 9;4:e012. doi: 10.48130/forres-0024-0009 (PMC11524237; doi:10.48130/forres-0024-0009)
Supplement: Supplementary file 1 — Supplementary data to this article can be found online. [file forres-0024-0009-S1.zip › 10.48130_forres-0024-0009-Suppl-FigureS5.pdf]

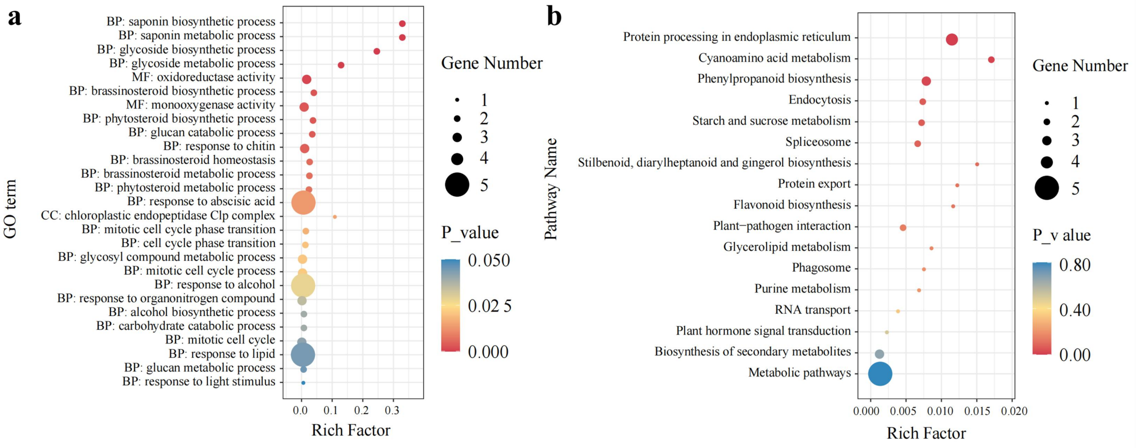

Supplementary Figure S5 Functional enrichment analysis of GWAS candidate genes. a: GO enrichment analysis of candidate genes, where the size of the points represents the number of genes, and the color of the points indicates significance level. BP, CC, MF respectively represent biological process, cellular component, and molecular function. b: KEGG enrichment analysis of candidate genes, where the size of the points represents the number of genes, and the color of the points indicates significance level.
